# Family flock size and age–sex structure of indigenous village chickens

**DOI:** 10.1002/vms3.70026

**Published:** 2024-09-17

**Authors:** Takele Taye Desta, Oli Wakeyo

**Affiliations:** ^1^ Wolaita Sodo Agricultural, Technical, Vocational, Education and Training College Wolaita Sodo Ethiopia; ^2^ Present address: Department of Biology, College of Science and Mathematics Education Kotebe University of Education Addis Ababa Ethiopia; ^3^ Present address: College of Agriculture and Environmental Science Arsi University Asela Ethiopia

**Keywords:** effective population size, family flock size and structure, high flock turnover, indigenous village chickens, smallholder farmers

## Abstract

**Background:**

Indigenous village chickens (IVCs) significantly contribute to rural development. There is considerable variation in family flock size and age–sex structure of IVCs.

**Objectives:**

This study reports the family flock size, age–sex group structure, and demographic history of IVCs.

**Methods:**

This work involved a cross‐sectional study conducted using face‐to‐face general informants’ individual interviews with 119 smallholder farmers in highland and lowland agroecological zones.

**Results:**

The average family flock size of the sampled households was small (mean = 7.28, range: 1–38). Female birds (hens and pullets) represented the largest proportion of the family flocks (50.2%). The proportion of chicks (26.4%) and pullets (14.4%) was lower than that of hens (35.8%), which creates a considerable challenge in producing replacement breeding flocks. Similarly, the proportion of cockerels (9.1%) was lower than that of cocks (14.3%). The average cock‐to‐hen ratio (one cock to seven hens) was better than the commonly recommended proportion (1 cock to 8–10 hens). As a result, smallholder farmers have not faced the problem of producing infertile eggs. The estimated effective population size (Ne) of 4.02 and the corresponding inbreeding coefficient of 0.12 at the family flock level show that to some extent inbreeding may occur at the family flock level; besides, the estimated Ne represents 55.2% of the census size. However, inbreeding could be offset by the high rate of flock turnover and uncontrolled mating among scavenging birds.

**Conclusions:**

Family flocks contain a few birds, which may lead to consanguineous mating. Inbreeding is, however, considerably reduced by uncontrolled breeding among birds that share a common scavenging ground. The driving factors behind the low proportion of chicks and juvenile birds and the family flock size need to be further investigated to improve the contribution of local chickens to rural livelihoods.

## Graphical abstract

Family flocks that are kept by small‐scale farmers inherently contain few birds. Regardless of small flock size, family flocks show a low rate of inbreeding due to high flock turnover. Multigenerational birds that are forming the family flock make the scavenging chicken production self‐sustaining system.

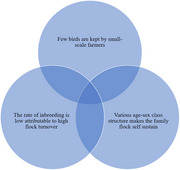

## 1 INTRODUCTION

Indigenous village chickens (IVCs) display a wide array of flock structures and compositions (Desta, 2021a, 2021b, 2021c; Mtileni et al., 2012). IVC flocks constitute multi‐generational birds (overlapping generations) and various age–sex (including functional classes of hens) groups. A few birds usually establish and maintain the family flock. By consensus, the family flock is composed of up to 100 adult birds (FAO, 2004). This cut‐off point has been set due to the absence of intensive production of IVCs and specialization, and the challenge encountered in keeping a large flock of IVCs under an extensive production system. However, even this small family flock consists of various age and sex groups. This multi‐generational flock structure has made the production of IVCs a self‐reliant small‐scale rural enterprise. In IVC production systems, flock size and composition vary significantly among seasons and households (Desta, 2019; Desta & Wakeyo, 2013; Tadesse & Tesfay, 2013). Family flocks are mainly composed of female birds, that is, hens and pullets of various ages and functional groups (Hailemichael et al., 2016).

Knowledge of flock size and composition is crucial for developing appropriate breeding plans and improving management practices, including the identification of an appropriate mating ratio and demographic history, that is, effective population size and rate of inbreeding. Flock structures signify smallholder farmers’ breeding objectives. The flock structure represents the number of hens, cocks, pullets, cockerels, and chicks. In IVCs, chicks represent the largest proportion of family flocks (Mahoro et al., 2017; Mujyambere et al., 2021).

A low rate of inbreeding, high genetic diversity, an intricate web of gene flow, a comparable proportion of breedable males and females, and advantageous mutations increase effective population size (Naskar et al., 2012). Likewise, reduced rates of co‐ancestry, non‐skewed reproductive success, and weak directional selection upsurge effective population size (Caballero & Toro, 2002; Sundström et al., 2004). Smallholder farmers' breeding objectives are broad and could be fulfilled by keeping phenotypically diverse birds; concurrently, this practice maintains an effective population size (Nielsen et al., 2005). A high effective population size reduces inbreeding rate, which enhances the performance of family flocks. In outbred IVCs, the effective population size is usually large (Desta, 2015); for example, it is significantly higher than the critical value of 100 proposed by Foose (1983). However, FAO (2010) states that Ne greater than 50 is a sufficiently large effective population size. According to Henson (1992), the acceptable rate of inbreeding per generation ranges from 1% to 2%. At least critical Ne should be maintained, especially for livestock species with short generation intervals (Simianer, 2000), which include chickens with 1‐year generation intervals (Fulton, 2009). This study reports the size and age–sex group structure of smallholder farmers' family flocks and estimates the effective population size and the associated rate of inbreeding.

## 2 MATERIALS AND METHODS

This section describes the study site, methods of data collection, types of data collected, and the statistical analysis made to summarize and interpret the data.

### 2.1 The study sites

The study site, the Wolaita zone, belongs to the culturally diverse and multilingual region of southern Ethiopia and is located between 6.4° and 7.1° N latitude and 37.4° and 38.2° E longitude. The Wolaita zone has a total area of 3982 km^2^. The elevation of the Wolaita zone ranges from 1200 to 2950 m above average sea level (masl). According to the customary classification method adopted in Ethiopia, the Wolaita zone is divided into three elevation‐based agroecological zones: kolla or lowland (35%, <1500 masl), woina dega or mid‐highland (56%, 1500–2400 masl), and dega or highland (9%, >2400 masl). The light rainy season usually lasts from March to May and the heavy season from July to October. The mean annual rainfall is 1014 mm. The mean daily temperature is 19.9°C; however, the daily temperature ranges from 17.7°C in July to 22.1°C in February and March (Wolaita Zone Plan Department, 2020). Wolaita is one of the regions that supply the significant proportion of live birds to terminal (central) markets in Addis Ababa.

### 2.2 Sampling methods

A cross‐sectional study was conducted in two districts of the Wolaita zone: Damot Gale, which represents the highland region, and Humbo, which represents the lowland region. Data were collected through one‐on‐one and face‐to‐face general informants’ interviews. The study was conducted in six representative rural villages selected from two agroecological zones (three from each agroecological zone) after consulting the district's livestock extension advisory service. The selected villages in the highland and lowland regions show a clearly marked ecological contrast. Respondents from each village were selected using systematic sampling method from a master's list of the farmers living in each village. Accordingly, after dividing the total number of farmers in the list by 20 (the sample size of each village), the first farmer was randomly selected using a lottery method from the first‐class interval. The remaining 19 farmers were subsequently selected at fixed intervals, equal to the class interval. A semi‐structured questionnaire, that was pre‐tested using 10 farmers, was administered to 119 small‐scale farmers (20 in each village, except Taba, from which 19 farmers were interviewed).

### 2.3 The studied traits

Farmers were approached to report their demographic characteristics and size and the age and sex structure of their flock. The agroecological zone and gender and education status of the respondents were used as explanatory variables.

### 2.4 Statistical analysis

The effective population size (*N_e_*) and rate of inbreeding (Δ*F*) were calculated using the number of adult male and female birds, following Wright's (1931) equations. Accordingly, the Ne and Δ*F* were calculated using Equations (1) and (2), respectively.

(1) $$N_{e}=\frac{4\times N_{m}\times N_{f}}{N_{m}+N_{f}}$$

(2) $$\Delta F=\frac{1}{2N_{e}}$$

where $N_{e}$ is the effective population size, $N_{m}$ is the number of cocks, $N_{f}$ is the number of hens, and ΔF is the inbreeding coefficient.

The effective population size can also be calculated using the change in the rate of inbreeding and generation intervals using Equation (3), following Muasya et al. (2013).

(3) $$Ne=\frac{1}{2\Delta Fy\;\times \;L}$$

where L is the generation interval.

Equation (4) is used to estimate the disparity between the effective population size and the census (actual) size.

(4) $$NeR=\frac{Ne}{N}$$

where NeR is the ratio of the effective‐to‐census size, and N is the census size.

Statistical analyses were made using IBM SPSS Statistics 23 (IBM Corp., 2015) and R (R Core Team, 2016). Descriptive statistics, χ test, *t* test, and Mann–Whitney U test were used to analyze the data.

## 3 RESULTS

This study reports the findings of a cross‐sectional study regarding demographic characteristics of extensively managed IVCs.

### 3.1 Flock structure

The results show that cocks represented 14.3% of the sampled flocks, with associated 35.8% of hens, 9.1% of cockerels, 14.4% of pullets, and 26.4% of chicks, and these proportions exhibit statistically significant differences (*χ* ^2^ = 23.663, *df* = 4, *p*‐value = 9.331e‐05). Among the respondents, 98.3% kept at least a hen, 64.4% maintained at least a cock, 47.5% kept at least a pullet, 35.6% owned at least a cockerel, and 39.0% possessed at least a chick. Although the proportions of cocks and hens were significantly different (*χ* ^2^ = 7.0634, *df* = 1, *p* = 0.007868); however, this was not the case for the proportions of cockerels and pullets (*χ* ^2^ = 1.7041, *df* = 1, *p*‐value = 0.1918). The results show that hens and pullets (excluding chicks, for which sex was not identified) represented the highest proportion (50.2%) of the family flock. The proportion of hens and pullets is significantly higher than the proportion of cocks and cockerels, that is, 23.4% (χ^2^ = 9.7587, *df* = 1, *p*‐value = 0.001785). These findings show that, unlike commercial birds, the IVC flock structure is not composed of a uniform age and sex group. The sampled household family flock size shows a high coefficient of variation (78.1%) indicating the higher dispersion of data points around the mean. Summary statistics for the various age and sex groups of family flocks are presented in Table 1.

**TABLE 1:** Descriptive statistics of age–sex class structure of the sampled family flock.

Flock size and age–sex group	Summary statistics					
	Mean	Median	Mode	Standard deviation	Minimum	Maximum
Flock size	7.28	6	3	5.69	1	38
Cocks	1.62	1	1	1.11	1	5
Hens	2.65	2	2	2.11	1	18
Cockerels	1.83	1	1	1.17	1	5
Pullets	2.21	2	1	1.58	1	10
Chicks	4.93	4	3	3.09	1	16

The Mann–Whitney U test of independent samples shows that only the number of chicks (*p* = 0.002) and pullets (*p* = 0.009) show a statistically significant difference between the highland and lowland regions. However, the number of cocks, hens, cockerels, chicks, and functional groups of hens did not show statistically significant differences apportioned to agroecology (see also Table 2 for the outcome of *t* test statistics). The average flock size was significantly higher in the lowland (8.41 ± 5.500) compared to the highland region (6.15 ± 5.693) (Table 2). The formal education level of the respondents had a statistically significant effect on the number of chicks (*p* = 0.008), hens (*p* = 0.012), pullets (*p* = 0.036), and laying hens (*p* = 0.015). Accordingly, as the formal education level of the respondents is advanced, the number of chicks, hens, pullets, and laying hens increases. Family size had a statistically significant effect on the number of hens (*p* = 0.018) and specifically on layers (*p* = 0.043). There was a positive correlation between the respondents’ family size and flock size (*r* = 0.286, *p* = 0.002).

**TABLE 2:** Flocks’ age–sex and functional group differences between lowland and highland regions.

Age–sex class	*t*	*df*	Significance (two‐tailed)	95% confidence interval of the difference	
				Lower	Upper
Flock size	2.188	116	**0.031**	−4.295	−0.213
Cocks	0.487	74	0.628	−0.386	0.635
Hens	0.482	114	0.631	−0.969	0.590
Cockerels	0.571	40	0.571	−0.529	0.945
Pullets	1.402	54	0.167	−1.428	0.253
Chicks	0.628	44	0.534	−2.632	1.382
Laying hens	0.066	76	0.948	−0.581	0.621
Hatching hens	1.279	31	0.210	−0.216	0.943
Brooding hens	0.754	49	0.455	−0.197	0.434
Free‐living hens	1.622	27	0.116	−1.264	0.148

*Note*: The *p*‐value for the statistically significant difference observed between agroecology is shown in bold.

Family flock size shows a statistically significant high positive correlation with the sampled family flock age–sex group and the number of laying, incubating, hatching, and brooding hens (range: 0.523–0.791, *p* = 0.000). However, flock size did not show a statistically significant correlation with the number of free‐living hens (*r* = 0.198, *p* = 0.303). Respondents’ age only shows a statistically significant positive correlation with the total number of hens (*r* = 0.186, *p* = 0.046) and specifically laying hens (*r* = 0.224, *p* = 0.049). The detailed correlation analysis of flock size and age–sex class of the family flock and functional groups of hens are presented in Table 3.

**TABLE 3:** Correlation between the number of age–sex class and functional groups of hens in the family flock.

Age–sex class/functional groups of hens		Cocks	Hens	Cockerels	Pullets	Chicks	Laying hens	Hatching hens	Brooding hens	Free‐living hens
Flock size	Pearson correlation	**0.539**	**0.720**	**0.523**	**0.752**	**0.791**	**0.619**	**0.646**	**0.682**	0.198
	Significance (two‐tailed)	0.000	0.000	0.000	0.000	0.000	0.000	0.000	0.000	0.303
Cocks	Pearson correlation		**0.454**	0.384	**0.427**	0.082	**0.422**	**0.743**	**0.411**	−0.158
	Significance (two‐tailed)		0.000	0.064	0.011	0.666	0.002	0.000	0.016	0.472
Hens	Pearson correlation			**0.494**	**0.611**	**0.358**	**0.863**	**0.825**	**0.711**	0.313
	Significance (two‐tailed)			0.001	0.000	0.015	0.000	0.000	0.000	0.099
Cockerels	Pearson correlation				**0.498**	−0.081	**0.693**	0.540	0.429	0.463
	Significance (two‐tailed)				0.003	0.757	0.000	0.086	0.053	0.151
Pullets	Pearson correlation					0.344	**0.672**	**0.657**	**0.567**	0.086
	Significance (two‐tailed)					0.149	0.000	0.002	0.006	0.802
Chicks	Pearson correlation						**0.561**	0.229	**0.509**	0.098
	Significance (two‐tailed)						0.007	0.585	0.000	0.788
Laying hens	Pearson correlation							**0.845**	**0.614**	0.264
	Significance (two‐tailed)							0.000	0.001	0.342
Hatching hens	Pearson correlation								0.445	0.144
	Significance (two‐tailed)								0.197	0.817
Brooding hens	Pearson correlation									0.070
	Significance (two‐tailed)									0.838

*Note*: Statistically significant correlations (*p*‐value ≤ 0.05) are shown in bold.

### 3.2 The proportion of functional groups of hens

Four functional groups of hens were identified: laying, incubating and hatching, brooding, and free‐living. The descriptive statistics for the different functional groups of hens that were kept in the flock are presented in Table 4. As expected, among hens, the layers represented the largest proportion of functional groups. Among the respondents who kept at least a hen, 40.8% owned at least a layer, 26.7% at least a brooding hen, 17.3% at least an incubating and hatching hen, and 15.2% owned at least a free‐living hen (multiple responses exist). The reported proportions of functional groups of hens exhibited a statistically insignificant difference (χ^2^ = 16.314, *df* = 3, *p* = 0.0009775). There was a lower percentage of incubating and hatching hens compared to brooding hens because the fledging of chicks takes a longer time compared to the incubation and hatching of eggs, which takes 21 days.

**TABLE 4:** The summary statistics of different classes of hens that make up the sampled family flocks.

Groups of hens	Range	Mean	Standard deviation	Median	Mode
Laying hens	0–10	1.11	1.340	1	1
Incubating and hatching hens	0–5	0.37	0.721	0	0
Brooding hens	0–3	0.55	0.732	0	0
Free‐living hens	0–4	0.48	0.952	0	0

### 3.3 Cock to hen ratio

In the sampled family flock, the cock‐to‐hen ratio ranged from 1–2 to 1–15, with a mean and standard deviation of 1:6.97 ± 1:2.28, a median of 1:6.5, and a mode of 1:6. Although the mean value of the cock‐to‐hen ratio significantly deviated from that is expected under natural conditions, that is, 1:1, a cock kept for a group of hens was greater than the ratio usually recommended by extension advisory services (1 cock: 8–10 hens). Nonetheless, the number of cocks was lower than that of the hens, which could be attributed to the high offtake rate of cocks. For example, according to informal discussion with some of the respondents, cocks are frequently disposed of through selling, slaughtering, or misaligned killing by predators. Cocks usually have a wide home range, which exposes them to predators and theft. This disparity in sex ratio increases the rate of inbreeding by reducing the effective population size. Among the explanatory variables, agroecology showed a statistically significant impact on the cock‐to‐hen ratio (*t* = 2.532, *df* = 117, *p* = 0.013, 95% CI: 0.2252–1.8412). Accordingly, the mean and standard deviation of the cock‐to‐hen ratio in the highlands were 1:7.49 ± 1:2.66 (standard error of the mean = 0.3081), whereas, in the lowland region, it was 1:6.46 ± 1:2.08 (standard error of the mean = 0.2682).

### 3.4 The effective population size and the associated rate of inbreeding

The effective population size and the number of cocks and hens used as breeding flocks were calculated for the sampled family flocks only; hence, they may not be conclusive enough for the entire population of Wolaita chickens. The effective population size of the family level was 4.02. The corresponding inbreeding rate of 0.12 may pose a threat to the genetic vigour of the family flocks. However, this could be offset by high flock turnover and uncontrolled breeding among scavenging flocks (Desta & Wakeyo, 2024). Therefore, these results should be interpreted in the light of this insight. Since the generation interval of chickens is about 1 year, estimating Ne using Equation (3) yields a similar result to that of Equation 1; therefore, we have not analyzed the data using Equation (3). Owing to the disparity in the number of breedable cocks and hens, that is, the number is higher in hens, and this lowers the effective population size. The relative Ne calculated using Equation (4) represents only 55.2% of the census size.

## 4 DISCUSSION

In Ethiopia, strangely, family flock size is small (5.7; Central Statistical Agency of the Federal Democratic Republic of Ethiopia [CSA], 2018), for example, compared to sibling developing countries, such as Uganda (2–113; Ssewannyana et al., 2008), South Africa (1–10; Yusuf, 2014), Sudan (31.18; Berima and Ishag, 2015), and Nigeria (13.9; Yakubu, 2010). This small flock size might have held back farmers from adopting technologies that enhance the productivity of family flocks, and not to earn better income and engage in gainful employment. This small flock size also leads to a waste of resources while delivering vaccinations that are usually packed for a moderate number (∼100) of chickens. Small flock size also increases market transaction costs while collecting a few numbers of birds and eggs from family flocks that are kept by small‐scale farmers living scattered in rural areas (Conglin et al., 2023).

Ethiopia is an alpine country with highly rugged topographies that can significantly restrain the movement of scavenging flocks, resulting in limited intermixing of flocks, which in turn may cause some degree of localized inbreeding and fine‐scale genetic stratification (Desta, 2015). However, inbreeding could be offset by the strangely high rate of flock turnover attributable to the devastating impact of the highly infectious Newcastle disease virus strain, that is, virulent avian paramyxovirus type 1 circulating in Ethiopia (Bari et al., 2021; Damena et al., 2016), uncontrolled breeding, and intricate web of gene flow. It might be due to the flock structure and topography‐induced barriers that Ethiopian chickens show a remarkable level of genetic stratification (review by Desta, 2015). Conversely, the IVCs of sibling developing countries do not exhibit genetic stratification (Lyimo et al., 2014; Muchadeyi et al., 2007; Osei‐Amponsah et al., 2010).

Compared to sibling developing countries (e.g., Berima and Ishag, 2015; Yakubu, 2010), the cock‐to‐hen ratio found in this study shows a smaller proportion of cocks in the family flocks. However, the ratio is higher than that recommended by the FAO (2004), that is, 1 cock to 8–10 hens to produce fertile eggs. Commercial farms also recommend a mating ratio of 1 cock:10 hens (Molapo & Kompi, 2015), which is lower than the findings of this study. However, possessing an equal number of cocks and hens, as in the case of idealized natural populations, significantly increases the effective population size and genetic diversity of chickens. This low proportion of cocks may often occur in IVCs because cocks are frequently slaughtered and sold. After all, cocks fetch better prices owing to their meatiness and consistent use in ritual practices (Conglin et al., 2023). This disparity in the cock‐to‐hen ratio also exists in natural populations, such as junglefowl, where hens outnumber cocks in a group of flocking birds (Collias & Saichuae, 1967).

Cocks, as guardians of the flocking birds, might be more frequently killed by predators. Cocks are usually slaughtered for family consumption and to welcome guests of honour. Farmers should be aware that a few cocks are required for breeding. Famers have adopted an open village chicken breeding system in which a cock can mate with hens that are belonging to various family flocks but scavenging together. Unlike hens, cocks do not provide multiple products and services, for example, regarding sex‐limited traits such as laying, and are not involved in incubating and hatching eggs or brooding chicks (Desta, 2019). All these inherent limitations have resulted in a higher slaughter rate of cocks. Cocks fetch better prices owing to their large size, magnificent plumage colour, and pattern and are often used for ritual commitments because male gods are commonly worshiped compared to female gods (Desta, 2021b). Although costly, aggression in cocks is important to evade predators. However, due to the small size of the family flock and the inadequate number of competent, aggression might be mild. Regardless of this, attributable to high flock turnover, aggression may occur frequently between newly introduced birds and established members of the flock to establish a new pecking order. Cocks uniquely show vivid plumage and morphological structures, such as comb and wattle, attributable to the impact of sexual selection, but this vividness exposes them to predator sightings and attacks.

Unexpectedly, this study found a smaller number of chicks than hens. A similar finding was reported by a large‐scale cross‐sectional study conducted in Ethiopia's highlands (Hailemichael et al., 2017), a sample survey conducted at the national level in Ethiopia (Central Statistical Agency of the Federal Democratic Republic of Ethiopia [CSA], 2018), and a study conducted in South Africa (Mtileni et al., 2012). This flock structure constrains flock breeding practice by reducing the number of replaceable flocks and is detrimental to the resilience of the family flock. A high preference for the consumption of eggs from local chickens and the associated high price may reduce the number of eggs set for incubation, thereby reducing the number of chicks. Moreover, chicks are disproportionately killed by predators and die because of disease, adverse weather conditions, and suboptimal management (discussion by Mtileni et al. [2012]). There is also a tendency to synchronize hatching with the dry season; therefore, if studies are conducted during the off‐dry season, the proportion of chicks might be found to be less. This flock structure shows that insufficient effort has been made to increase the size of the family flock. Smallholder farmers (especially men) may not give sufficient attention to auditing the number of chicks; hence, the number of chicks may have been underreported.

Family flock size shows statistically significant variations among the sampled households and agroecological zones. Flock size is significantly affected by the recent history of disease outbreaks (Awan et al., 1994), the contribution of IVCs to smallholder farmers' livelihood, prevailing demand for poultry and poultry products, and the suitability of the production system for rearing IVCs. There was a considerable proportion of free‐living hens, which might be represented by retired hens that might be in transition between laying cycles or recently fledged chicks and have not started laying. Maternity is a costly trait, and hens become free‐living because they require sufficient time to acquire physiological rest.

Flock size is larger in the lowlands; this might be because the highland region is more suitable for crop production attributed to enough rain, so crop production has a comparative advantage over IVC production. IVCs are also detrimental, especially to newly established crops (Desta & Wakeyo, 2013). The off‐take rate of chickens in highland regions may be high because of the dense human population. Unlike a significant number of siblings in developing countries, chickens are the only poultry species kept by smallholder farmers in Ethiopia.

Small flock sizes lead to high rates of inbreeding due to genetic drift (Mahoro et al., 2017). The estimated Ne was less than the census size. Ne approaches the census size when the number of breedable male and female birds is equivalent and makes the largest proportion of the flock. Nevertheless, even under an equal proportion of male and female birds, Ne may not be equal to the census population because hens choose which cocks to mate with and because a dominant hierarchy may exist among cocks, which favours the dominant cock to mate frequently and produce more chicks. By keeping a constant cock‐to‐hen ratio, Ne increases proportionally as the census size increases.

## 5 CONCLUSION

IVCs contain a multigenerational flock structure in which birds of various ages, sexes, and functional groups are reared together. The family flock size is uniquely small in Ethiopia, and in‐depth studies are required to identify the driving factors behind this small flock size. A smaller proportion of chicks, for example, than hens, is an unexpected finding, and it could be the subject of future studies. The Ne of the studied chicken population, although less than the census size, shows that the Wolaita chicken population is not under threat. To improve the production and productivity of the family flock, and the contribution of IVCs to rural livelihood, there is a need to increase the size of the family flock and the number of farmers engaged in the rearing of IVCs.

## AUTHOR CONTRIBUTIONS

Takele Taye Desta and Oli Wakeyo were involved in the data collection. Takele Taye Desta analyzed the data and drafted the manuscript. OW commented on the draft of the paper.

## CONFLICT OF INTEREST STATEMENT

The authors declare no conflicts of interest.

## ETHICS STATEMENT

The involvement in his study was strictly voluntary, and all information gathered from the participants was kept confidential and was used only for research purposes. Informed verbal consent was obtained from all the respondents.

## Data Availability

The data will be available from the corresponding author upon reasonable request.
